# Effects of Resveratrol, Curcumin and Quercetin Supplementation on Bone Metabolism—A Systematic Review

**DOI:** 10.3390/nu14173519

**Published:** 2022-08-26

**Authors:** Alessio Danilo Inchingolo, Angelo Michele Inchingolo, Giuseppina Malcangi, Pasquale Avantario, Daniela Azzollini, Silvio Buongiorno, Fabio Viapiano, Merigrazia Campanelli, Anna Maria Ciocia, Nicole De Leonardis, Elisabetta de Ruvo, Irene Ferrara, Grazia Garofoli, Valentina Montenegro, Anna Netti, Giulia Palmieri, Antonio Mancini, Assunta Patano, Fabio Piras, Grazia Marinelli, Chiara Di Pede, Claudia Laudadio, Biagio Rapone, Denisa Hazballa, Alberto Corriero, Maria Celeste Fatone, Andrea Palermo, Felice Lorusso, Antonio Scarano, Ioana Roxana Bordea, Daniela Di Venere, Francesco Inchingolo, Gianna Dipalma

**Affiliations:** 1 Department of Interdisciplinary Medicine, University of Bari “Aldo Moro”, 70124 Bari, Italy; 2 Department of Interdisciplinary Medicine—Intensive Care Unit Section, University of Bari Aldo Moro, Piazza G. Cesare 11, 70124 Bari, Italy; 3 PTA Trani-ASL BT, Viale Padre Pio, 76125 Trani, Italy; 4 Implant Dentistry College of Medicine and Dentistry Birmingham, University of Birmingham, Birmingham B4 6BN, UK; 5 Department of Innovative Technologies in Medicine and Dentistry, University of Chieti-Pescara, 66100 Chieti, Italy; 6 Department of Oral Rehabilitation, Faculty of Dentistry, Iuliu Hațieganu University of Medicine and Pharmacy, 400012 Cluj-Napoca, Romania

**Keywords:** polyphenols, flavonoids, bone metabolism, osteoporosis, diabetes, bone tumours, periodontitis, microbiota, oralbiotica, oralbiotics

## Abstract

Phenolic compounds are natural phytochemicals that have recently reported numerous health benefits. Resveratrol, curcumin, and quercetin have recently received the most attention among these molecules due to their documented antioxidant effects. The review aims to investigate the effects of these molecules on bone metabolism and their role in several diseases such as osteopenia and osteoporosis, bone tumours, and periodontitis. The PubMed/Medline, Web of Science, Google Scholar, Scopus, Cochrane Library, and Embase electronic databases were searched for papers in line with the study topic. According to an English language restriction, the screening period was from January 2012 to 3 July 2022, with the following Boolean keywords: (“resveratrol” AND “bone”); (“curcumin” AND “bone”); (“quercetin” AND “bone”). A total of 36 papers were identified as relevant to the purpose of our investigation. The studies reported the positive effects of the investigated phenolic compounds on bone metabolism and their potential application as adjuvant treatments for osteoporosis, bone tumours, and periodontitis. Furthermore, their use on the titanium surfaces of orthopaedic prostheses could represent a possible application to improve the osteogenic processes and osseointegration. According to the study findings, resveratrol, curcumin, and quercetin are reported to have a wide variety of beneficial effects as supplement therapies. The investigated phenolic compounds seem to positively mediate bone metabolism and osteoclast-related pathologies.

## 1. Introduction

Phenolic compounds (PFs) are naturally occurring phytochemicals comprising more than 8000 molecules, with plant growth and protection functions acting as fungicides, natural pesticides, and food inhibitors [1].

**Figure 1:**
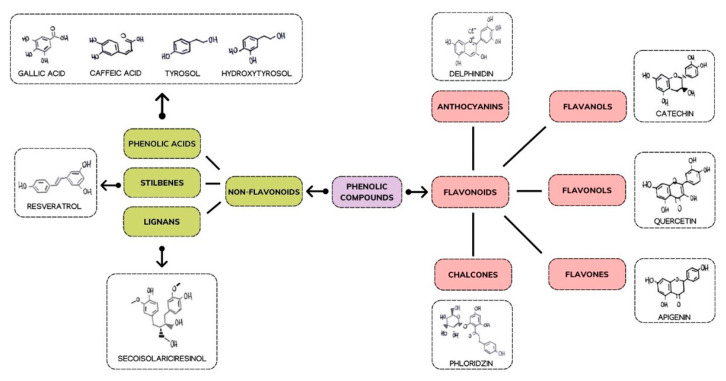
Schematic classification of phenolic compounds with chemical structures.

PFs possess an aromatic structure with multiple hydroxyl groups. Their classification is based on the type of bonding between carbon atoms, and the position and the number of hydroxyl groups they exhibit [2] (Figure 1).

PFs are divided into two subclasses based on the number of carbon atoms: flavonoids and non-flavonoids.

Flavonoids are characterized by having 15 carbon atoms. Flavonoids can be classified into flavones, isoflavonoids, flavandiols, flavonols, flavanonols, flavanols, and anthocyanins chalcones anthocyanins and chalcones, the latter of which is characterized by some structural variations from the former [3].

**Figure 2:**
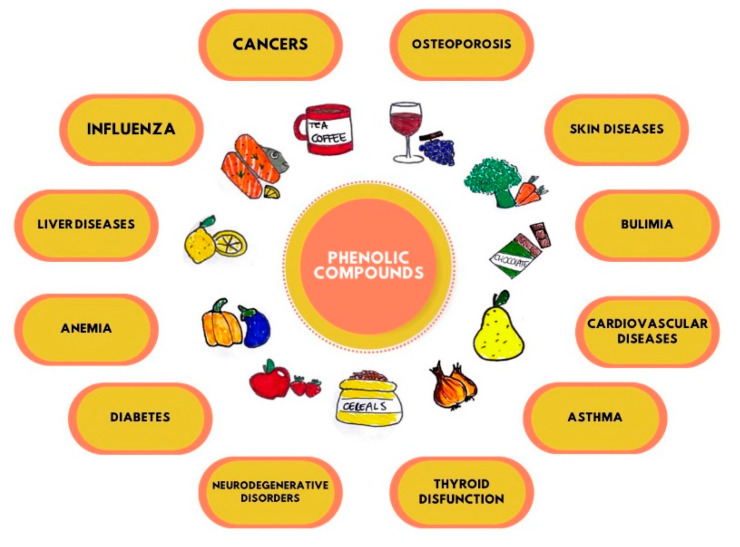
Representation of therapeutic properties of phenolic compounds on multiple human diseases.

Non-flavonoids have heterogeneous structures that comprehend phenolic acids with only one aromatic ring, and stilbenes and lignans have two rings of carbon atoms linked together by different chains [4]. Among the PFs, Quercetin (Q), Resveratrol (RSV), and Curcumin (CU) belonging to the flavanols, stilbenes, and curcuminoids family, respectively, have been studied in the treatment of multiple human diseases [5,6,7,8,9,10,11,12,13,14,15]. Recently, the natural PFs aroused an interest in scientific research according to multiple properties for human health. PFs are recognized to have many properties: antioxidant, anti-tumour, antibacterial, antiviral, anti-inflammatory, anti-ageing, antifungal, and antithrombotic, as well as having beneficial effects on diabetes, cardiovascular diseases, neurodegenerative diseases, and modulating bone-specific metabolism in resorption bone diseases [2,16,17,18,19,20,21,22,23,24,25]. Recent studies have shown that PFs have epigenetic effects. They influence the expressive capacity of the genetic heritage, a key role in cancer therapy and in controlling the onset of degenerative diseases [26,27,28,29] (Figure 2).

**Figure 3:**
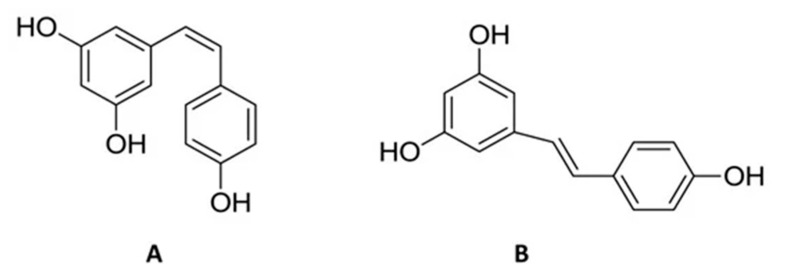
Chemical formula of CIS-Resveratrol (**A**) E TRANS-Resveratrol (**B**).

The RSV (trans-3,5,4′-trihydroxystilbene) occurs naturally in red fruits such as raspberries, fermented grapes, plums, mulberry seeds, nuts, peanuts, red wine, blueberries, and in plants such as *Polygonum cuspidatum* and flowers [30]. Commonly, RSV is represented by two isomeric forms, trans and cis-resveratrol. The second form is more photosensitive and unstable. It is soluble in substances such as dimethyl sulphoxide and ethanol and it is not water-soluble [30,31] (Figure 3). In literature, RSV reported several benefits due to its anti-inflammatory, anti-tumour, antioxidant, antithrombotic, anticoagulant, antiviral, and immunostimulant properties. In addition, it seems to produce a protective effect and benefits against neurodegenerative diseases by preventing cognitive retardation [31,32,33,34]. In addition, it seems to produce a protective effect and benefits against neurodegenerative diseases and neuroinflammation by preventing cognitive retardation [35]. Orally administered RSV activates sirtuin 1 (SIRT1), which improves the energy production from the mitochondrial activity and inhibits fat accumulation acting on both glucose and fat metabolism, producing the effects of a low-calorie diet [36,37,38]. RSV regulates intestinal homeostasis, serving as a target for the intestinal microbiota and thus having antioxidant effects [39,40,41].

RSV is credited with the property of interacting with both the gut microbiota and their metabolites, such as short-chain fatty acids and gut wall lipids, by improving the clinical manifestations of metabolic syndrome, which is a rampant disease in modern societies, manifested by abdominal obesity, hyperlipidaemia, hypertension, diabetes, and cardiovascular diseases [42,43,44].

**Figure 4:**
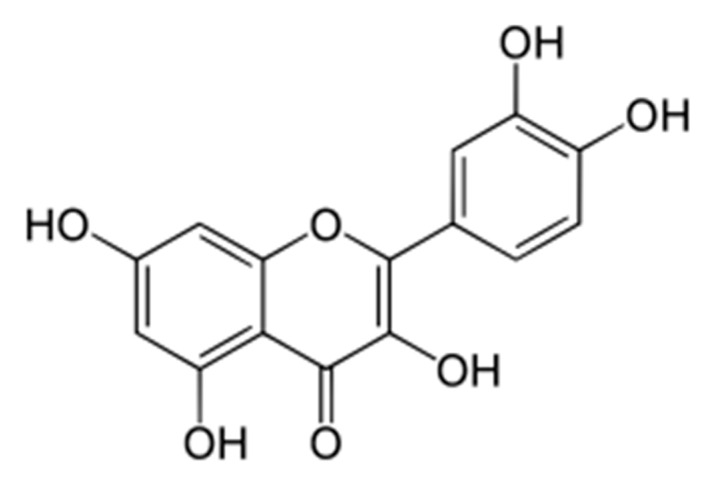
Chemical formula of Quercetin.

The Q is one of the most abundant phenolic compounds in foods such as fruits, vegetables, nuts, and seeds, and it is particularly found in capers, red onions, cabbage, and organic tomatoes (Figure 4). It is found in nature in various forms: in vegetables and in the structure of Q-3-O-glucoside which acts as a pigment for coloured fruits. The Q has the same form that was mentioned previously when it is in foods, while dietary supplements mainly contain the free state of the Q, the aglycone. The benefits of the Q have been discussed in relation to cardiovascular diseases, diabetes, inflammation, asthma, viral infections, and cancer prevention [45]. According to an Italian regulation in dietary supplements, it is recommended that for human supplementation, a daily intake of up to 200 mg is needed. In contrast, in fruit and vegetables that are consumed daily, it is about 1000 mg. It has been estimated that a consumer of high amounts of fruits and vegetables can also incorporate pure Q into their daily diet, in the amount of 1250 mg of Q. The Q can perform antioxidant, anti-inflammatory, antiviral, anti-obesity, and antidepressant actions and prevents cancer, diabetes, asthma, hypertension, and cardiovascular diseases [46]. Plants are not the most bioavailable sources of the Q. The source with the highest bioavailability of the Q for humans is found in red onions [47]. After ingestion, the Q interacts with salivary proteins and forms soluble aggregates, then arrives in the stomach, where the acidic pH conditions break down the phenolic acids, followed by the process of glycosylation by a hydrolase in the small intestine, thereby obtaining the aglycone. It then passes through the liver, which is metabolized and circulates as metabolites, which can be tested in blood and urine for the presence of bioactivity. An effective dose for blood pressure reduction is 500 mg when it is in its free form [45].

**Figure 5:**
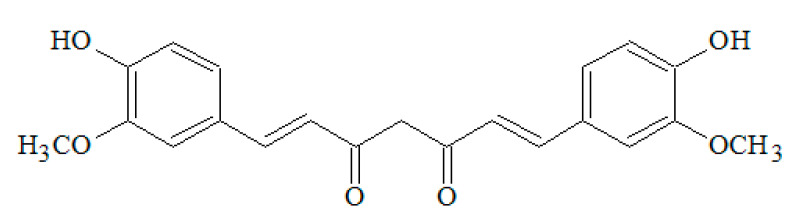
Chemical formula of Curcumin.

The CU is the biologically active part of turmeric, which belongs to the ginger family (the Zingiberaceae) (Figure 5). The CU is obtained by drying the root of a *Curcuma longa*. This Asian plant presents a high number of calories as it contains 70% carbohydrates and a few lipids and proteins [48]. The CU is recognized as a safe substance according to GRAS (General Recognition and Safety) by the American FDA (Food and Drug Administration). Several types of research have shown that the CU can be administered to adults at up to 8000 mg/day (16 capsules per day) with no toxic effects occurring. Clinical studies have shown that CU has therapeutic properties for dyspepsia and peptic ulcers [49]. The administration from one (1) to four (4) capsules daily with meals is recommended to control the inflammatory processes that occur during food intake and digestion. The CU is an effective anti-inflammatory in both its acute and chronic phases. The mechanism of action consists of a cyclooxygenase’s inhibition, usually obtained with nonsteroidal anti-inflammatory drugs, and the lipoxygenases’ inhibition, commonly produced by steroidal anti-inflammatory drugs. Moreover, the CU is very effective in rheumatoid arthritis treatments, also with a local administration [50]. In addition, Cianciulli et al. reported that a CU had a potential effective action on inflammation control and modulation in neurodegenerative diseases [51].

**Figure 6:**
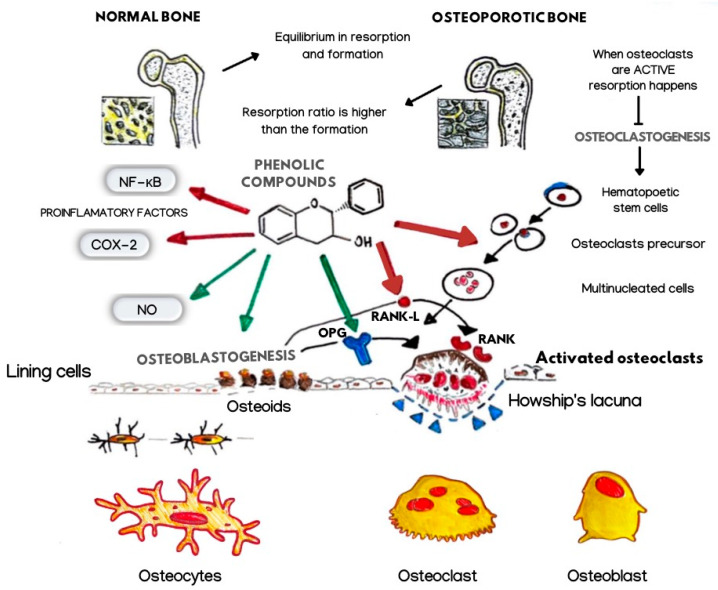
Representation of the therapeutic properties of phenolic compounds on bone metabolism.

Recent studies have shown the antiproliferative activity of CU that blocks both the production and action of the tumour necrosis factor (TNF), proving that it is a useful adjuvant in anticancer therapies and in controlling the proliferation of drug-resistant tumours [52]. The CU stimulates the adrenal cortex to produce endogenous steroids and blocks hepatic cortisone production [53]. This molecule can perform an antioxidant action by inhibiting the release of free radicals, slowing the cell degenerative processes [53]. The CU is also recognized to perform an immune-stimulating action due to the presence of a polysaccharide and antispasmodic portion at the hepatic and gastroenteric levels. In hepatic digestive functions, the CU has choleretic and cholagogic properties [54]. The CU is chemically sensitive to heat, light, and a pH > 5. In the literature, the piperine extracted from black pepper has been combined with increased bioavailability. There are ongoing studies on the liquid drop form, which would seem more bioavailable [54]. Recent studies on the Q, the CU, and RSV have confirmed a positive influence in controlling bone metabolism by reducing osteoclastic activity or activating osteoblastic activity [55]. The bone metabolism alterations are frequently related to the resorption process either in the form of generalized osteolysis, such as osteoporotic fractures in old age and post-menopause subjects, or focal ones, especially at the level of joints in rheumatoid arthritis and bone metastases [56]. Therefore, the level changes of sex hormones such as oestrogen and testosterone, growth factors, and increased adiposity in the bone marrow, hematopoietic cells, and aged cells could influence the bone resorption processes, producing an increased osteoclast activity stimulated by RANKL [55,57,58,59,60,61,62,63,64] (Figure 6).

In addition, the RSV, the CU, and the Q are present in the host circulation as glucuronides. Their deconjugation is regulated by biological mutations in the bone and the presence of β-glucuronidase (GUSB), which recognizes them as substrates and catalyses them into the active aglycone; this is required for anti-osteoclastogenic activity and the consequent protective effects on bone resorption [65,66]. In patients with a menopause/oestrogen deficiency (GUSB), the PFs can have protective effects on the skeleton structure, which require the active aglycone form [67,68].

At sites with increased cellular turn-over in bone metastases or skeletal accretion, the bioavailability of PFs that are glucuronidated in bone may be increased. Consequently, the active aglycone increases, producing anti-osteoclastic effects [69,70].

The therapeutic properties of PFs are consumption-dependent and thus, dose-dependent. The PF is known to be absorbed and metabolized by the intestine into glucuronides and sulphates. The active aglycone form is not present in blood plasma [71]. The anti-inflammatory effects of Q-3-O-glucuronide and Q-3′-O-sulphate, derived from Q-glucuronide and catalysed by GUSBs in macrophages, have been studied. The dietary administration of these substances when activated in macrophages would control age-related inflammatory processes by reducing their effects and bone metabolism [43]. Some studies reported that by administering RSV associated with strontium ranelate, mesenchymal stem cells (MSCs) activate toward the osteogenic lineage with an increase in serum alkaline phosphatase and bone alkaline phosphatase concentrations. However, no change was found in osteocalcin that was specific to bone turnover and procollagen [72]. The RSV also reported benefits on bone metabolism by the activation of SIRT1. In cells where SIRT1 is present, RSV administration controls fat metabolism, glucose metabolism, and gluconeogenesis processes, preventing diabetes and cardiovascular disease and improving cell turnover by slowing the ageing process [38,73]. In a study of diabetic patients, who manifest bone fractures frequently, there was an improvement in bone density after the daily administration of it at 500 mg. In addition, RSV, by reducing the onset of diabetes, also reduces the possibility of occurrence of metabolic bone disorders [72,74]. Evident outcomes of the improvement of bone density following RSV administration were obtained in patients with significantly lower calcium and 25-hydroxy vitamin D concentrations at the outset and in patients who abused alcohol use [75]. Some research has shown that RSV administration increases osteoblastic activity and slows the processes of osteoporosis [72]. Moreover, the RSV combined with platelet concentrates, such as concentrated growth factors (CGF), blocked the osteonecrosis of the jaw due to the administration of bisphosphonates, particularly from zoledronate [76,77,78,79]. Another beneficial effect of RSV at the bone level is it preventing the occurrence of *Staphylococcus aureus* osteomyelitis by blocking thrombotic events caused by damage to neutrophils produced by the Panton-Valentine leukocidin toxin (PVL) [80]. The limitations of the therapeutic applications of phenolic compounds are due to their poor stability, which reduces the bioavailability of phenolic compounds in the body. The RSV blood concentrations are usually too low because it is rapidly absorbed and eliminated by the body in the liver [81,82].

Recently, many studies have been interested in prolonging the bioavailability of PFs by contrasting their rapid elimination. An analysis of the data showed that in patients taking RSV, combined with vitamin D with and without calcium, there was an improvement in bone density accompanied by a 7.2% reduction in C-terminal telopeptide (CTX) marker of bone resorption [83,84,85,86]. Besides the systemic effects, the PFs could present local benefits on soft and hard oral tissues, preventing gingival recessions and peri-implant inflammation and bacterial invasions, which are the common leading causes of dental implant failure. Biomedical research investigates the coupling between materials to achieve a combination of biomaterials functionalised with natural molecules to evaluate their possible beneficial effects on biological tissues [87]. Recent studies by Manuel Gomez-Florit et al. showed how coating titanium surfaces with quercitrin improved the integration of dental implants due to its antioxidant, anti-inflammatory, and antimicrobial capabilities. Nanococcal surfaces coated with Q were shown to reduce bacterial adhesion and increase the adhesion of human gingival fibroblasts [88]. Alba Cordoba et al. suggest applications in orthopaedics, demonstrating that the surface modification of the titanium substrate with polyphenolic molecules (specifically taxifolin and Q) promotes the differentiation of human umbilical cord-derived stem cells (hUC-MSCs) into osteoblasts, increasing alkaline phosphatase activity and calcium deposition and optimizing the expressivity of osteogenic markers, thus conferring the osteoporosis monitoring activity to the surface [89]. Therefore, this finding could promote studies in implantology to promote osseointegration.

The present study aimed to investigate the effects of RSV, the CU, and the Q on bone metabolism and to evaluate the therapeutic potential of natural substances that could provide valuable support to traditional therapies.

## 2. Materials and Methods

### 2.1. Search Processing

The current systematic review followed the PRISMA and International Prospective Register of Systematic Review Registry procedures (full ID: CRD42022347033) [90]. The PubMed, Web of Science, Google Scholar, Scopus, Cochrane Library, and Embedded databases were examined for articles in-line with the study topic from January 2017 to 3 July 2022, with an English language limitation. The search strategy was created by combining terms relevant to the study purpose. The following Boolean keywords (Table 1) were applied: (“resveratrol” AND “bone”); (“curcumin” AND “bone”); (“quercetin” AND “bone”).

### 2.2. Inclusion Criteria

The reviewers worked in pairs to assess all relevant studies according to the following inclusion criteria: (1) clinical trials on humans and in vitro studies on human cells; (2) open-access publications that other investigators can read for free; (3) research that investigated the positive effects of the intake of these three phenolic compounds on bone metabolism and their application in certain clinical conditions such as osteoporosis, bone tumours, and periodontitis. In addition, their use on the titanium surfaces of prostheses used in orthopaedics was investigated. Studies that did not meet the above criteria were excluded.

### 2.3. Data Processing

Two independent reviewers (F.P. and A.C.) assessed the quality of the included studies using specified criteria such as selection criteria, methods of outcome evaluation, and data analysis. This enhanced ‘risk of bias’ tool additionally includes quality standards for selection, performance, detection, reporting, and other biases. The inclusion criteria were used to discover possibly published full-text publications. Any differences were settled through conversation or collaboration with a third researcher (F.V).

### 2.4. Risk of Bias Assessment Methodologies

The assessment of the studies risk of bias (RB) was performed in full accordance with the OHAT risk of bias guidelines for humans and animals studies and the in vitro studies tool of the United States National Toxicology Program (NTP). The RB evaluation was assessed separately for in vivo and in vitro studies. The RB of in vivo studies followed the criteria: confounding bias, detection bias, confidence in the outcome, reporting bias, and other biases. The RB of the in vitro studies considered the following parameters: experimental condition, blinding during the study, incomplete data, exposure characterization, outcome assessment, reporting, and other biases. The risk of bias output was categorized as adequate, unclear, or inadequate and were finally classified as low risk of bias if a minimum fraction of 5/7 positive parameters were detected. Otherwise, the articles were considered as high risk of bias studies.

## 3. Results

### 3.1. Characteristics of Included Articles

**Figure 7:**
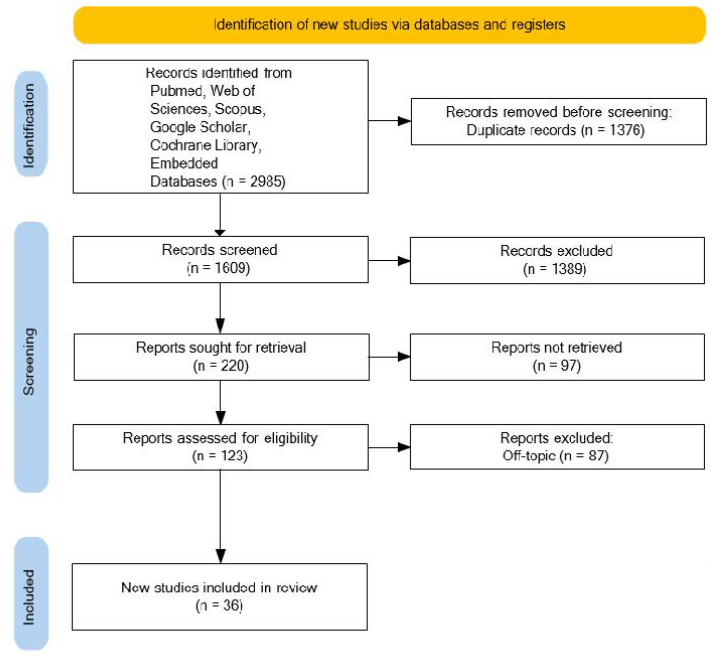
PRISMA flowchart diagram of the inclusion process.

A total of 2985 publications were identified in six databases, including PubMed (664), Web of Science (842), Scopus (1429), Google Scholar (2), Cochrane Library (48), and Embedded (0), obtaining 1609 records after the duplicates were deleted (1376). The title and abstract analyses resulted in the exclusion of 1389 articles. The remaining 220 records were recovered, producing 123 reports for which the authors determined eligible. Eighty-seven publications were excluded from the discussion because they did not meet the inclusion criteria. The evaluation includes a total of 36 publications for qualitative analysis (Figure 7).

### 3.2. Risk of Bias of the Included Studies

#### 3.2.1. In Vitro Studies

**Figure 8:**
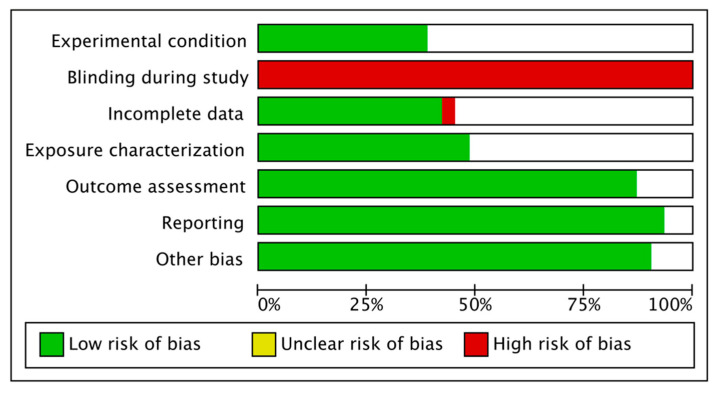
Synthesis of the RB assessment of the in vitro studies.

**Figure 9:**
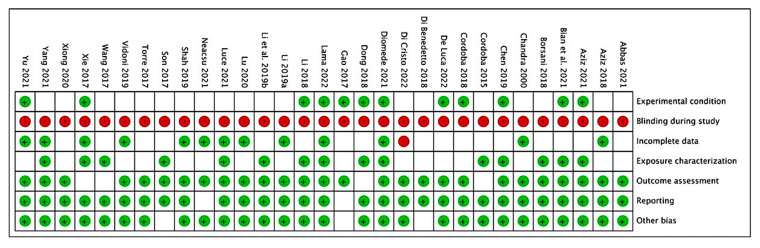
Detail of the RB assessment of the in vitro studies included [76,89,91,92,93,94,95,96,97,98,99,100,101,102,103,104,105,106,107,108,109,110,111,112,113,114,115,116,117,118].

The risk of bias assessment of the in vitro studies is reported in Figure 8 and Figure 9, with a total of 31 included papers. A total of 10 articles showed a low risk of bias (32.25%), considering the heterogeneity of the model design and the methodologies. The most frequent bias percentages were blinding (100%), experimental condition (62%), incomplete data (59%), exposure characterization (52%), outcome assessment (12%), other biases (9%), and reporting (6%).

#### 3.2.2. In Vivo Studies

**Figure 10:**
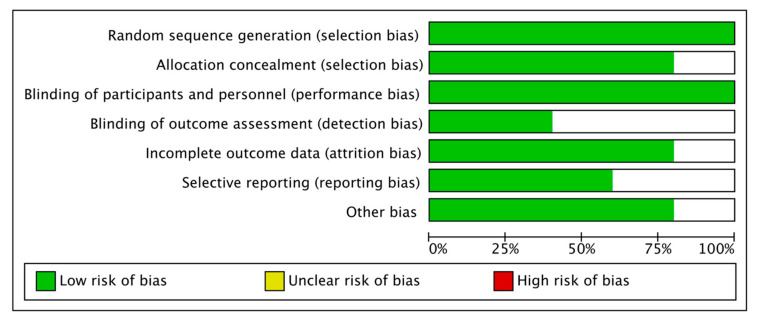
Synthesis of the RB assessment of the in vivo studies.

**Figure 11:**
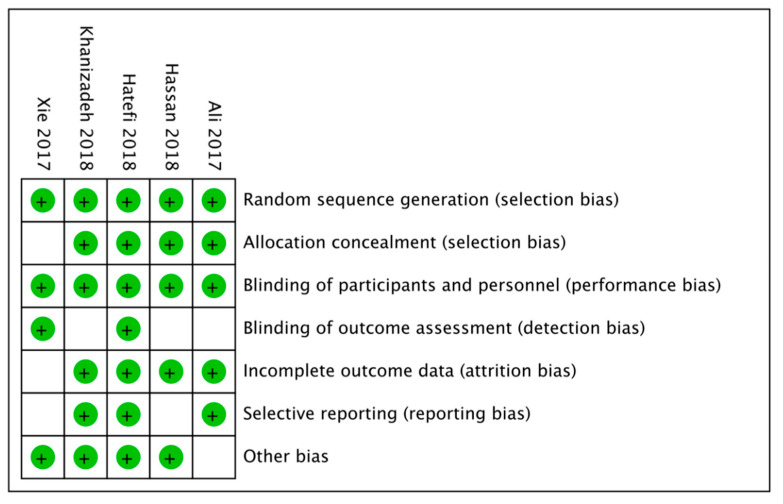
Detail of the RB assessment of the in vivo studies included [57,104,119,120,121].

The risk of bias assessment of the in vitro studies is reported in Figure 10 and Figure 11, with a total of five included papers. A total of four articles showed a low risk of bias (80%). The most frequent bias percentages were blinding of outcome assessment (60%), selective reporting (40%), incomplete outcome data (20%), allocation (20%), other biases (20%), blinding of participants and personnel (0%), and random sequence generation (0%).

### 3.3. Bone Metabolism Modulation

In several studies, the flavonoids that represent the main subclass of PFs are effective in promoting osteogenic activity and counteracting osteoclastic activity by acting on different signalling pathways [122] which include: Wingless-INT/β-catenin, insulin-like growth factor [123], bone morphogenetic protein [124], runt-related transcription factor 2 (Runx2) [125], and Osterix [126]. Furthermore, some PFs are also called phytoestrogens as their structural similarity with some mammalian oestrogens makes them able to bind to ERs α and β, thus acting as hormonal analogues (with agonist and antagonistic effects based on the tissue) [127].

#### 3.3.1. Resveratrol

The RSV seems to work as a selective oestrogen receptor modulator (SERMs) and a mixed agonist/antagonist [128]. The RSV’s anti-inflammatory and antioxidant properties may impact bone metabolism; RSV’s pharmacological potential and safety profile may be used as a treatment for bone problems [129]. In some studies (Table 2), bone mass was improved by RSV, showing that RSV stimulates OBs or inhibits osteoclastic resorption [130]; this suggests its possible use in the treatment of osteoporosis with numerous advantages.

In the study of Shah et al., the potential osteogenetic activity of RSV during different stages of OB differentiation was investigated. Three stages of OB differentiation were distinguished. There is an early proliferative period in which cells proliferate and express fibronectin, collagen, and osteopontin; the matrix maturation period is characterized by the arrest of the cell cycle and the beginning of OB differentiation with an increase in alkaline phosphatase (ALP) and collagen [91]. Matrix mineralization constitutes the final stage of differentiation and it is characterized by hydroxyapatite synthesis; the bone consists of 60% hydroxyapatite, 10% water, and 30% organic component [131]. Many researchers have studied the mechanisms that regulate the osteogenic effect of RSV using osteogenic and OB cell lines [130]. The RSV acts on ALP activity and DNA synthesis, thereby stimulating the proliferation and differentiation of MC3T3-E1 cells; however, this action has been blocked by tamoxifen, an antioestrogen, thus inferring that RSV activity is influenced by an oestrogen-dependent mechanism [132]. According to a study on rat calvarial OB cells [133], RSV stimulates OC gene expression independently of SERM. The immortalised human foetal OB cells 1.19 (hFOB) have few ERs (less than 200) capable of binding 17 β-estradiol and translocating it into the cell nucleus [134,135]. In a study by Chakraborty et al., 2013, partial agonist activity on the α-isoform oestrogen receptor was observed [128,133].

There are few studies of RSV action on hFOB. Shah et al. [78] studied the anabolic effect of RSV through oestrogenic-independent mechanisms using a safe concentration of RSV of 62.25 µM with a cell viability of 69.3% (Table 1). The apoptosis occurred at higher concentrations such as 125, 250, and 500 µM. According to preliminary research, therapeutic doses of 500 nM and 1 M were selected; at these concentrations, ALP activity was unaffected [91]. However, the total protein content and mineralization were significantly stimulated according to time and dose-dependent mechanisms. RSV treatments showed a significant increase in calcification within 14 days (Table 2). Therefore, the present study highlighted the effect of RSV in the late mineralization phase of hFOB cells, suggesting its anti-osteoporotic action through a possible non-oestrogenic signalling mechanism [91]. As reported in Abbas et al. RSV seems to be implicated in the RANKL signalling mechanism. The results of their research, performed in vitro and in silico, have demonstrated that RSV binds to RANKL (Table 2). RSV interacted with a considerable number of amino acids of RANKL, thus blocking it. The fusion of RSV with the RANKL protein proved to be highly efficient, thus making RSV an inhibitory substance of RANKL with biological activity against bone loss [92]. The same conclusion was reached by Zhao et al.; this study demonstrated that high doses of RSV (40 and 80 mg/kg) inhibit bone resorption and decrease osteoclastic activity in the RANKL/RANK pathway [136]. The use of RSV at high doses interfered with the RANKL/RANK/OPG (osteoprotegerin) pathway by blocking RANKL (ligand) in the phase of the beginning of osteoclastogenesis with an activity similar to that of OPG [137], which is a decoy receptor for RANK-L. OPG blocks RANKL at the initial stage preventing the onset of osteoclast activation [78,138]. Also, once NF-kB is activated in mononuclear cells, the transcription of peptides and interleukins (IL-1, IL-6, IL-8) will begin, which are very important in the inflammatory response, as well as for up-regulating the expression of TNF as a pro-inflammation gene [139,140]. During the ageing process, we generally have a progressive pro-inflammatory status. Evidence is showing, more and more, that pro-resorptive cytokines such as (TNF-α) tumour necrosis factor-alpha and interleukins (IL-1, IL-6) could potentially be interfering with age-related osteoporosis [141,142]. The use of RSV significantly decreases the concentration of IL-6 and TNF-α. Also, a decrease in the concentration of TGF-β1 was observed in rats who underwent an ovariectomy [136,137]. In addition, the actine cytoskeleton needs to be reorganised because it is responsible for the osteoclasts’ spreading, mobility, and bone resorption. With the help of actin-rich adhesions called podosomes, osteoclasts connect to the bone to carry out their activity. The M-CSF/integrin signalling system controls the construction and dynamics of the actin cytoskeleton. An in vitro study (Table 2) demonstrated that RSV reduced the actin ring formation [92], inhibiting the osteoclasts’ adhesion to the bone. The RSV’s effect on osteoarthrosis (OA) has been investigated over the years. Abed studied the impacts of RSV on OB affected by OA through an in vitro study of human tibial plateaus [143]. SIRT1 expression is reduced in subchondral tibial bone affected by OA when compared with OB from the healthy bone. In contrast, leptin has a high concentration in OA and is reduced in a healthy OB. In this study, it was shown that RSV has a protective action in OA because it stimulates the expression of SIRT1 and reduces that of leptin through the phosphorylation of Erk1/2 [143]. Vidoni et al., in 2019, investigated the impact of RSV on human mesenchymal gingival cells (HGMSCs) and specifically studied the role of autophagy during the OB differentiation of RSV-activated MSCs. Autophagy plays a crucial role in the cellular remodelling that occurs during the differentiation of MSCs. The mTORC1 pathway inhibits the autophagy process, but the AMPK pathway activates it. RSV acts by stimulating the transcription of the osteogenetic factor, RUNX2 [93]. At 1 µM, RSV induces the differentiation of HGMSCs into osteoclasts in a synergistic action with factors that stimulate osteogenesis; however, it is toxic when the concentration exceeds 10 µM. In synergy with osteogenesis-inducing factors, RSV activates the pro-autophagic AMPK-BECLIN1 pathway in HGMSCs [93]. The applications of this study are interesting for regenerative therapy in maxillary and mandibular bone defects through the use of scaffolds containing HGMSCs, RSV, and autophagy modulating factors [93,144]. In 2018, Li et al. looked at how oxidative stress impairs the hBM-MSC’s ability to differentiate into OBs and how SIRT1 contributes to this [94]. The SIRT1 pathway actively contributes to the antioxidant and osteogenic processes in MSCs. SIRT1 and other antioxidant enzymes (superoxide dismutase 2, catalase, and glutathione peroxidase 1) are naturally upregulated in oxidative stress; this in vitro study [94], they showed that RSV could boost SIRT1’s antioxidant protective effects on early differentiated hBM-MSCs via improving the matrix mineralization and the OB gene expression. The natural compounds polydatin (a glucoside of RSV), found in many different kinds of fruits and vegetables, and RSV, found in red wine, have been studied thoroughly due to their anti-inflammatory and antioxidant properties [95]. The very low bioavailability and rapid metabolism of RSV were significant flaws that made polydatin a better therapeutical substitute, even though the role of this substance is still enigmatic [95]. MSCs extracted from SONFH have been employed in cell-based treatments despite their significantly reduced osteogenesis capacities. Polydatin noticeably increased alkaline phosphatase activity in human bone marrow mesenchymal cells (HBMMCs) and induced the formation of calcium nodules and the expression of other cellular markers that manifested in different stages of the differentiation process [96]. Moreover, the OC is a molecule explicitly manifested in the latest stages of osteogenic differentiation [96]. Although polydatin significantly increased osteogenic differentiation, the underlying processes causing this are unknown [95]. According to in vitro research, RSV stimulates the differentiation of OB cells, which produces bone and its progenitors [96]. RSV and polydatin have both important antioxidant qualities, but polydatin has a better capacity to control oxidative stress. Finally, recent research showed that polydatin increased the endurance of BMSCs, shielding them from oxidative stress despite suppressing proliferation and causing death via catenin signalling at high doses in human osteosarcoma cells [95]. So, this makes polydatin a possible therapy for the treatment of osteoporosis (Table 2).

#### 3.3.2. Curcumin

Curcumin (CU) is the main polyphenolic component of Curcuma, a powder made from the rhizome of *Curcuma longa* of the Zingiberaceae (ginger) family [145]. The CU has undergone several studies for its healing properties as it has anti-inflammatory, antioxidative, and anticancer effects, demonstrating beneficial effects in the therapy of numerous cardiovascular diseases, cancers, and systemic inflammation [146]. Stem cells belong to a population of undifferentiated cells with the capacity for self-renewal and differentiation. MSCs are essential for differentiating bone, cartilage, and adipose tissues [147]. The MSCs and other cells from different organ tissues undergo apoptosis and degeneration because of oxidative stress, which aggravates inflammatory, metabolic, and degenerative illnesses [97]. Kim et al. reported that MSCs were separated and treated using a combination of CU, ginger, vitamin D, L. rhamnosus, and Boswellia extract (NS-J) to assess their ability to respond to inflammatory stress. The investigation showed that the natural integrators, especially ginger and curcumin, protected MSCs from oxidative stress and that the various NS-J components worked together synergistically to do the same for human MSCs. The potential osteogenic differentiation of MSCs using calcium deposition in grown OBs was also investigated, examining the outcomes of the supplements used, individually and synergistically. The use of the NS-J formula, synergically, is demonstrated to be more effective in increasing the osteogenic potential of MSCs. Regarding the chondrogenic effects, NS-J treatment significantly increases the synthesis of type II collagen, the main constituent of the functional articular cartilage of chondrocytes. Moreover, this chondrogenic potential also prevails under inflammatory conditions, i.e., the peroxisome proliferator-activated receptor (PPAR-d) pathway is associated with the NS-J complex, therefore, the NS-J complex has anti-inflammatory effects [148]. Therapy with CU (0.1–10 μmol/L for 2 days) promotes MSC proliferation and modifies the extracellular matrix (ECM) characteristics [149]. At high concentrations, however, CU shows cytotoxicity to healthy human OBs, with a significant reduction in cell density after a 24-h treatment, making curcumin treatment dangerous and requiring more investigation [150]. In a study by Yang et al., MSCs were continually exposed to various concentrations of CU, and the regenerative properties of MSCs were investigated by analysing the maintenance, proliferation, migration, and adipogenic and osteogenic differentiation of human bone marrow-derived mesenchymal cells (hBM-MSCs). The hBM-MSCs were handled with 0.01–100 μmol/L CU for seven days. Regarding the toxic effect of CU on hBM-MSCs, there were no toxicities in, in vitro maintenance with CU amounts of 10 μmol/L or less. According to the dosage of CU that was delivered, the proliferation that hBM-MSCs induced differed, i.e., with CU concentrations of 0.01–1 μmol/L, the proliferation significantly increased, which was halved or null with concentrations of 10 μmol/L and 5 μmol/L. Indeed, the results indicate that concentrations of 5 and 10 μmol/L induced cell apoptosis and reduced cell division in hBM-MSCs. These concentrations would be recommended for the therapy of some cancers but may induce harmful effects on adult stem cells, such as hBM-MSCs. The findings of this study showed that treatment with 5 µmol/L CU reduced the spread of matrix metalloproteinases (MMPs) in hBM-MSCs, enzymes that are involved in metastatic processes. MSCs are a part of the renewal of bone, cartilage, and adipose tissues. This study confirms that treatment with small doses of CU enhances osteogenic differentiation but inhibits the adipogenic differentiation of hBM-MSCs [98]. Son et al. studied curculactones A and B, two new elements obtained by the radiolysis of CU; this paper also confirmed that these two new compounds induce OB differentiation in MSCs [99]. The activity of the natural mix showed synergic activity when compared to the characteristics of the individual ingredients, and the beneficial effects of this were amplified. Therefore, the curcumin-based compound should help treat arthritis and osteoarthritis [148] (Table 3).

#### 3.3.3. Quercetin

Like RSV, Q also appears to affect bone metabolism, and the subsequent studies demonstrate its implication in different bone homeostasis regulation mechanisms (Table 3). Torre et al., in a 2020 study [100], evaluated the effects of pomace extracts rich in PFs (PRPE) extracted from two types of grapes (Arneis and Croatina) on the osteogenic differentiation of MScs. Each PRPE contains Q, rutin, gallic acid, caffeic acid, p-coumarin acid, and malvidin-3-glucoside. The MSCs were incubated with the two kinds of PRPE, and the effects on osteogenic differentiation of the PFs contained in them were studied using a RT-qPCR. It was observed that there was an increased expression of genes involved in osteogenic differentiation, such as BMP2 and Runx2 [100]; the differentiation of MSCs into OBs due to the increased expression of the Col1a1 and SPARC genes involved in the deposition of the ECM (ECM) occurred in the early stages of bone formation; a reduced transcription of mineralisation genes such as ALP and OC are expressed in the more mature stages of OB differentiation [100]. The PFs contained in the Arneis and Croatina PRPEs reduce free radicals and, therefore, the mitochondrial oxidative stress that occurs in the differentiation phase of MSCs in osteogenic cells, exerts a pro-osteogenic effect [100]. To investigate the impact of PFs on bone resorption caused by inflammation, the influence of this PRPE on the RANK gene, responsible for the expression of the factors underlying the osteoclastogenesis process, was evaluated by Torre et al. and it was found that in their presence in this gene was downregulated [100] (Table 4).

In 2021 Bian et al. studied the ability of Q to act on osteogenic differentiation through the H19/miR-625-5p axis [101]. H19 activates ALP and the transcription of BMP-2, RUNX2, and OC mRNAs, which positively affect the osteogenic differentiation of hBM-MSCs [101].

Q, therefore, acts on the H19/Mir-625-5p axis and activates the Wnt/β-catenin signalling pathway favouring the osteogenic differentiation of hBM-MSCs (Table 4) [151,152,153].

Bian et al. suggest that Q’s effective, non-cytotoxic dose is 1~2 μm [101].

### 3.4. Osteopenia and Osteoporosis 

Osteopenia is a metabolic disorder that consists of a reduction in bone density. If undetected and mistreated, the gradual loss of calcification can eventually result in osteoporosis (OP) and fractures. It is a significant and impactful public health issue, affecting more than 20 million elderlies in the US, especially postmenopausal (PM) women, and it causes about 1.5 million fractures, annually [154].

According to the predictions, the number of 50-year-old people in the US with osteopenia rose from 43.4 million in 2010 to 52.0 million in 2020 and will reach 57.2 million in 2030 [155]. Dual Energy X-ray Absorptiometry (DXA), which measures the quantity of mineralised bone in the skeleton (bone mineral density, BMD), is now used to evaluate the increased fracture risk. Based on World Health Organization (WHO) guidelines, patients are deemed to have osteopenia based on their DXA measurements, when their BMD *t*-score of the spine or hip ranges between −1 and −2.5. In contrast, osteoporosis individuals have a t-score that is lower than −2.5 [156].

Studies have shown that in postmenopausal women, the hormonal changes linked to ovarian function after menopause cause the majority of the alterations in bone density [157]. The oestrogen receptor (ER) and androgen receptor (AR) that are expressed in osteoblast and osteoclast progenitors and their offspring influence the role of oestrogens and androgens on bone mass. However, the target genes of these activities are yet unclear. Some of the effects of oestrogens and androgens on bones may be caused by direct binding to EREs or AREs. The protective impact of oestrogens on the preservation of cortical bone mass may be the consequence of ER signalling that is non-nuclear-started. Regardless of ligand binding, the ER in mesenchymal cells could encourage bone apposition from the periosteum in either sex. Additionally, oestrogens increase the longevity of OB and osteocytes while decreasing that of osteoclasts, limiting skeletal turnover [158]. Recent investigations show that the fallout of oestrogens and their deficiency in bone remodelling is partly caused by the stimulation of the RANK Ligand/system RANK/osteoprotegerin (OPG). The interaction of the RANKL with the RANK determines the transformation of mononuclear precursors in mature osteoclasts. In PM women, increased rates of RANKL are expressed by the osteoblasts due to the reduction in oestrogens, promoting bone resorption [159]. Although the incidence of osteoporosis is constantly rising, and there are many breakthroughs in pharmacotherapy, most patients are not appropriately treated. In managing OP, bisphosphonates, hormone therapy, selective AR, calcitonin, and vitamin D analogues are used. However, these compounds are linked to some adverse effects that restrict their therapeutic uses. Finding innovative therapeutic agents that can enhance the bone-forming capacity is urgently required. One of the most effective ways to prevent osteopenia is through proper nutrition [160]; the intake of fruits and vegetables that are high in antioxidants has been correlated to postmenopausal women’s lower risk of OP and loss of BMD, according to a recent study. Oxidative stress accelerates bone remodelling and bone mass loss, whereas antioxidants reduce oxidative stress and stop bone mass loss [161,162]. Natural remedies might be supplementary or alternative; among these antioxidant molecules, RSV, CU, and Q are the most investigated. The advantages of antioxidant intake on skeletal structures require further studies.

#### 3.4.1. Resveratrol

RSV has anti-inflammatory, antioxidant, and bone-preserving qualities that are universally recognised as advantageous to human health. These actions are intermediated by promoting sirtuin 1 (SIRT-1). It is a polyphenolic substance found in red wine, peanuts, grapes, and mulberries, and other food.

According to much research, RSV promotes the growth and differentiation of osteoblast cells in a culture and inhibits the development of OP in rats and osteonecrosis in rabbits [77,163,164,165]. Jing Li et al. studied the function of RSV, which can perform multiple bioactivities of antioxidants, on mesenchymal stem cells derived from human bone marrow (hBM-MSCs) of subjects affected by OP. HBM-MSCs can differentiate into several cell lineages: chondrocytes, adipocytes, osteoblasts (OB), and osteocytes. They are critical to having healthy bones, treating bone disease, and regeneration [166,167]. The RSV exhibits anti-inflammatory and anticancer properties, but the parts concerning the biological functions of hBM-MSCs and OP are not well known.

At 40, 80, and 100 μM, RSV inhibited adipogenesis and promoted the reproduction of hBM-MSCs in HOB, which OP might decrease. Especially at a concentration of 80 μM, RSV improved the mineralisation capability of hBM-SCs and accelerated the creation of mineralised nodules. So, it could be considered a potential therapy for treating patients with OP. Borsani et al. studied, in vitro, the antioxidant efficacy of RSV, with or without concentrated growth factors (CGF) on human OB, in therapy or not with bisphosphonates (BP). They investigated the benefits to counteract the adverse effects of the utilisation of BP, such as bisphosphonate-related osteonecrosis of the jaw (BRONJ), monitoring indicative markers like bone morphogenetic protein-2 (BMP-2) and OPG. The RSV promotes human OB growth and differentiation and, in combination with RSV and CGF, has a preserving function on human OB that is treated with BP, especially with zoledronate. These results are promising better to investigate the roles of RSV in vivo research [76] (Table 5).

#### 3.4.2. Curcumin

The CU is one of the natural foods that is most researched due to its low toxicity and numerous molecular uses. Recently, the effectiveness of CU on bone homeostasis has been investigated both in osteopenia and postmenopausal OP and acts essentially through two mechanisms. The first consists of inhibiting the NF-kappa B ligand (RANKL) pathway, suppressing the growth of osteoclasts (and promoting osteoclast apoptosis). The second mechanism exerts a preserving role on the skeletal architecture by repressing metalloproteinase-9 (MMP-9), linked to the dissolution of the extracellular matrix. RCTs demonstrate these issues in animal models and humans [168,169]. In rats with ovariectomy-induced menopause, CU ameliorates the serum biochemical markers of bone turnover and histomorphometric bone parameters, alone or in combination with alendronate. In women affected by osteopenia, oral supplementation with CU at 1000 mg/day improved their bone density after 12 weeks and 24 weeks of therapy, in terms of the attenuation of the ultrasound passage of the heel (−18.4% at week 12 and −21.0% at week 24). It increased the grey-scale median measurement in ultrasound images of the small finger and upper jaw (+6.9% and +3.8% at week 12, and +7.1% and +4.8% at week 24, respectively). In women affected by osteoporosis, combined therapy with CU at 110 mg/day and an alendronate at 5 mg/day for six months decreased their level of bone-specific alkaline phosphatase (BALP) and c-terminal cross-linking telopeptide of type I collagen (CTx), significantly, and increased their osteocalcin levels when compared to the baseline and alendronate group. In addition, the synergistic effect of CU and alendronate also improved the BMD in four zones (total body and hip, femoral neck, and lumbar spine) after 12 months of therapy when compared to the control group and alendronate group (Table 6). In all studies, CU has shown a high tolerability and safety profile without having adverse effects. Used in combination therapy, CU’s anti-inflammatory and antioxidant properties antagonise the typical negative consequences of diphosphonates, namely arthralgia, myalgia, and epigastralgia. However, clinical studies in humans, investigating CU’s influences on bone health are scant.

#### 3.4.3. Quercetin

The Q is a natural flavonoid with effects that counteract oxidation and diabetes and protect against weight loss. Recent information shows that in diabetic patients and those with uncontrolled diabetes, the incidence of OP and fracture risk is higher than the average person of the same age [170,171]. Studies have identified several main mechanisms that would explain how glucose metabolism determines the origin of osteoporosis in type 2 diabetes patients, i.e., the increase in fat mass has a toxic consequence on the differentiation of the mesenchymal cells of the bone marrow, leading to bone loss and adipogenesis. Adipose tissue produces adipokines that influence bone metabolism, such as adiponectin, which supports it, and leptin, which promotes it. The alterations in the secretion of adipokines and cytokines and increases in the number of triglycerides in the vessels are linked to a reduction in bone turnover markers and BMD or augmentation in fatty tissue in the bone marrow. Furthermore, arterial hypertension reduces BMD concerning the increased urinary excretion of calcium [172].

Hyperglycaemia could induce the production of advanced glycation end-products (AGEs), which negatively affect bone quality due to several mechanisms. For example, AGEs increase the action of osteoclasts and the production of sclerostin (which negatively regulates bone formation) [173]. Another mechanism is the altered regulation of the calcium-vitamin D-parathyroid hormone. Inadequate glycaemic control is related to excessive calcium loss, leading to a chronic secretion of PTH, which compromises BMD. Diabetic nephropathy, a common complication, causes bone fragility. In particular, it involves a specific metabolic disorder, uremic osteodystrophy (Chronic Kidney Disease-Mineral Bone Disorder, CKD-MBD) [173]. The Q, when administered orally to diabetic patients, affects the mineralisation biomarkers, like vitamin D, osteocalcin, and calcium. The Q leads to increased serum calcium and vitamin D through 3 mechanisms: (a) the activation of the TRPV6 gene, which codes for the production of 25 vitamin D hydroxylase [25(OH)D]; (b) by acting as an agonist on the 1.25 OH vitamin D receptor; (c) as an antioxidant on the small intestine cells. Serum calcium levels are also related to osteocalcin levels. Osteocalcin is a calcium and phosphate binding protein, such as sialoprotein and osteopontin, which plays a role in bone mineralisation by regulating mineral deposition and the size and quantity of hydroxyapatite crystals in the bone [174]. In a double-blind, randomised controlled study, Q was administered at 500 mg/day for two months to a subject affected by type 2 diabetes mellitus. After three months, the grades of osteocalcin, calcium, and vitamin D were evaluated. Applying Q in diabetic patients can raise the amount of Vitamin D and calcium in the blood. It can also modulate the bone mineralisation represented by the increase of calcitonin. So Q supplements could be helpful for diabetes type 2 patients fighting osteoporosis [121] (Table 7).

### 3.5. Neoplasms and Bone Tumours

One of the biggest causes of death in people, globally, is cancer. Still, most solid tumours are now largely treatable if detected and treated before it begins spreading because of the developments in diagnostics and treatment [175]. Blocking the spread of tumours is essential to extending the survival of cancer patients and reducing their growth. The primary methods to stop metastasis involve focusing on cancer cell invasion and motility [176]. Both primary and secondary cancers can develop in the bone microenvironment, which is a perfect breeding ground [177]. Osteosarcoma (OS) is a primary malignant tumour that mainly develops at the ends of long bones and is uncommon. Young male patients are virtually always affected. OS develops from early mesenchymal cells, which usually come from bone rather than soft tissue [178]. As a primary bone tumour, OS occurrence and progression are tightly correlated with the bone microenvironment. The growth of osteoid tissue and juvenile bone from mesenchymal cells is a characteristic of bone cancer that is known as OS [179]. A high-grade bone cancer with significant invasive potential is OS [180]. The OS metastasis, in particular, is a therapeutic challenge characterized by a poor prognosis [181]. Although OS treatment is about to develop, increasing patient survival has long been challenging [182]. B7-H3, GD2, and HER2 are three surface proteins expressed by OS cells that may have therapeutic value [183]. These proteins can be targeted with antibody-drug conjugates and/or adoptive cell treatments [184]. Furthermore, suppressing the immunosuppressive tumour microenvironment may enhance the latter strategy by assisting in overcoming the immunological checkpoint.

#### 3.5.1. Resveratrol

Resveratrol has been well studied for its in vitro and in vivo anticancer activities, making it one of the most promising naturally produced chemo-preventive medicines [185]. Ying Gao and Chunnian He (Table 8) report that human bone cancer cells show antiproliferative activity when they are exposed to RSV and when compared to the RSV monomer, while the RSV oligomers (dimers and trimers) had more potent anticancer effects and antioxidant activity [103]. The RSV’s ability to control glucose absorption and lactate generation may cause this benefit [186]. The tissue of the bone is dynamic and constantly models and remodels itself. GJIC plays major functions in bone production and remodelling between the osteocytes, the OB, and the osteoclasts [187]. An essential part of GJIC is connexin 43 (Cx43), which is necessary for intracellular communication, controlling cell growth and differentiation, and preserving tissue homeostasis [188]. Connexins, particularly Cx43, play a crucial role in the GJIC, which controls bone formation, differentiation, modelling, and remodelling. OB development, differentiation, survival, and apoptosis are regulated by GJIC. It is also reported that GJIC affects the ability for osteoclast development and resorption [189]. Furthermore, in response to anabolic agents and mechanical loads, osteocytes employ GJIC to coordinate bone remodelling. The Wnt/-catenin signalling system controls the essential development elements in the embryonic stage and homeostasis in the adult phase. RSV lowers U2-OS cell proliferation and triggers apoptosis, reducing cell invasiveness in vitro. In addition, according to the study results of Xie et al., target genes of the Wnt/catenin signalling pathway showed a decreased expression [104] (Table 8). They demonstrated that RSV promotes apoptosis and inhibits U2-OS cell motility, invasion, glycolysis, and proliferation. Widespread expression of the Wnt/-catenin signalling pathway may be seen in bone tissue and cells, and its abnormal activation is intimately linked to the development of OS. RSV may therefore inhibit the activity of the Wnt/-catenin signalling pathway and the production of genes that are related to it (E-cadherin, -catenin, c-myc, cyclin D1, MMP-2, and MMP-9) [104]. Recurrences and metastases are the primary reasons for the poor prognosis of OS, and the 5-year survival rate in patients with OS recurrence and lung metastasis is less than 20% [190]. Unfortunately, this unsettling picture is frequently the result of chemotherapy failure brought on by the chemo-resistance phenomena [191], in addition to the numerous side effects of toxicity chemotherapy drugs [192], which frequently result in tumour progression and recurrence. According to research by De Luca and colleagues [105] (Table 8), RSV suppresses the proliferation of OS. Cells induce OS cells to suffer and participate in the pAKT and caspase-3 pathways, inhibiting proliferation and increasing the proportion of apoptosis [105]. The reduced release of the pro-inflammatory interleukins, IL-6 and IL-8, contribute to RSV’s effects on motility decrease and inhibition of proliferation, respectively. When compared to findings obtained with RSV or chemotherapeutic drugs alone, researchers [105] saw a substantial reduction in tumour cell proliferation after their treatment with RSV-doxorubicin (DOX) or RSV-cisplatin (CIS). In particular, it was demonstrated that a significant decrease in cellular proliferation was already seen at lower concentrations of RSV-DOX and RSV-CIS when compared to the chemotherapeutic medication. Polydatin (3,4′,5-trihydroxystilbene-3-β-d-glucoside, PD), a naturally occurring glycosylate precursor of resveratrol, which is extracted from the *Polygonum cuspidatum* plant, substitutes the hydroxyl group of RSV with a glycosidic ring that is attached at position C-3. The biological properties of PD are altered as a result of this alteration, showing improved oral absorption and metabolic stability when compared to RSV. The evidence is that properly PD-treated nanofibers facilitate adhesion and promote osteogenic differentiation in both MSC and human OS cell types [193]. By blocking the PI3K/Akt and PDGF/Akt pathways, PD has been shown to have anticancer properties [194]. The studies of Lama et al. (Table 8) suggest that PD promoted the proliferation of mesenchymal stem cells (MSCs) while inducing cell toxicity in OS cells Saos-2 cells; therefore, Saos2 cells’ ability to increase lessened under the influence of PD, along with a reduction in cell invasion and migration [106]. By measuring the bone alkaline phosphatase (BAP) activity, Luce et al. (Table 8) identified the impact of PD on osteogenic differentiation. In human Saos-2 cells, ALP activity was employed as a measure of osteogenic differentiation, and Saos-2 cells that were treated with PD had significantly higher BAP activity than the untreated cells did. They also looked at how PD affected osteoblast-mediated mineralization [107]. After 48 h of research, PD caused a 25% reduction in cells and stimulated osteoblast development and mineralization. Due to its high prevalence of metastases, as well as its resistance to chemotherapy and radiotherapy, OS has a poor prognosis [195]. PD pretreatment may boost the radiotherapy’s efficacy. The outcomes demonstrated that the Saos-2 cells were not radiation-sensitive. However, the pretreatment of PD for 96 h dramatically enhanced the sensitivity of the cells to modest doses of radiation (2 Gy), causing a reduction in viability, although the percentage of viable cells remained similar when compared to the control cells [107]. Osteopontin (OPN) expression appears to be crucial for the health of osteoblasts. OPN is required for integrin v3-mediated cell signalling to modulate osteoblast development [196]. Even though many malignancies have a poorer prognosis when OPN is overexpressed, in OS, OPN expression is inhibited, which slows the differentiation of mesenchymal stem cells or immature osteoblasts into mature osteoblasts, resulting in immature osteoblastic-like cells [197]. The scientific community has a keen interest in the health benefits of polyphenolic compounds on living organisms. RSV can cause a dynamic process. Therefore, it will be essential to propose novel approaches for treating OS based on the concurrent treatment of OS with radiation and the administration of RSV on a biological basis, which will result from the following investigations. In addition, further advantages could be taken from an RSV-adjuvant supplement in form of an oral daily administration [72,74]. In addition, on diabetic rats, the study findings seems to suggest an increased new bone formation and osteogenesis associated to RSV when it is administered through an intraperitoneal administration [198].

#### 3.5.2. Curcumin

CU also was investigated for its anti-cancer properties on many forms of cancer [199]. However, natural CU’s low in vivo absorption has limited its medicinal uses. Curcuminoid analogues have been synthesized to enhance this deficiency in physiology and biological activities because of its partial solubility in water [200]. Our investigation led to the analysis of several studies that used this natural compound in the context of primary and secondary bone tumours, as reported in Table 9. The anticancer efficacy of (Z)-3-hydroxy-1-(2-hydroxyphenyl)-3-phenyl prop-2-en-1-one (DK1), a synthetic CU analogue, was examined in vitro in the 2018 study on two OS cell lines: U-2 OS and MG-63 [108]. The aggressiveness of these two lines varied, with U-2 OS being more aggressive due to its capacity to infiltrate and move more quickly than MG-63 could [201]. According to the findings, the DK1 analogue inhibited both cell lines in a dose-dependent manner, but it did so to a considerable extent in U-20 OS cells [108]. In addition, the results revealed that the mode of cell death involved apoptosis, as was already reported in the literature [120], but it specified that it occurred through a mitochondria-dependent pathway [108]. The enhanced expression of pro-apoptotic genes and proteins in U-2 OS cells, including caspase 9, caspase 3, Bax, and cytochrome-c, supported this observation [108]. In a later Malaysian investigation, DK1 underwent additional examination to confirm its anti-metastatic and antiangiogenic characteristics [109]. In the human OS cell lines, U-20 OS and MG-63, DK1 significantly decreased the proportion of migratory and invading cells. This study showed that DK1 significantly inhibited the ability of the cells to form tubes and prevented microvessels from sprouting in a dose-dependent manner. These conclusions were amply proved by the strong up-regulation of numerous genes (PLK3, PTEN, FOXO, GADD45A, and Caspase) that impede metastasis and the significant down-regulation of other genes and proteins (Endoglin, uPA, Serpin, IGFBP-2, and FGF) that promote metastatic activity [109]. Instead, the group, Lu et al., examined the anti-metastatic activities against OS of a different synthetic analogue of CU, the 1-ethyl-3,5-bis((E)-3,4,5-trimethoxybenzylidene)piperidine-4-one (L48H37) [110], which had already been found to have effects on various cancers [202]. They suggested that L48H37 contributes to the suppression of human OS cell invasion and migration by inhibiting uPA and the JAK/STAT pathway [110]. The primary focus of Li’s study was to assess CU’s potential as an inhibitor of the human adipose-derived mesenchymal stem cells (hADSCs)’ osteogenic development [203]. The study’s findings demonstrated that CU alters the expression of specific miRNAs, which are often involved in osteogenesis and bone production. In this Chinese work, CU elevates the miR-126a-3p expression, and the triggering effect prevents the WNT pathway from activating. Thus, stem cell osteogenic differentiation is suppressed in a dose-dependent manner. High doses of CU, according to the research, may result in a reduced rate of osteogenesis, weakening the bone and increasing its susceptibility to metastasise [203].

The focus of research in recent years has been on nanoparticles as anti-cancer therapeutic possibilities and the supplementation of natural substances. A hydroxyapatite-CU combination was suggested in the Romanian study from 2021 to assess and enhance antiresorptive characteristics in bone cancers. It has been demonstrated that hydroxyapatite-CU systems may kill bone tumour cells in a dose-dependent manner by inducing apoptosis, raising AMPK and ARRB1 levels, and causing a G2/M cell cycle. This substance targets cancer cells only while minimizing the adverse effects on non-neoplastic reserve cells [111]. At the same time, the synthetic copolymer, poly-alendronate-ialuronane-S-S-curcumin (ALN-oHA-S-S-CU) was tested for its anti-cancer effects in Dong’s in vitro investigation [112]. In a hydrophilic setting, this substance may self-assemble into micelles. The disulphide link is broken in the tumour’s cytoplasmic microenvironment by reduction processes when the micelles penetrate the tumour through the active target, CD44; this results in drug release and tumour growth inhibition. Also, when using 3D models in this investigation, the molecule penetrated considerably further [112]. Additionally, Wang’s team looked into the anti-OS properties of artificial polymeric nanoparticles that are coated with curcumin (CU-NPr), which significantly overcame the difficulties of a free CU [113]. This approach prevented the metastatic OS 143B cells from proliferating, migrating, and invading, lowering the expression of c-Myc and MMP7 at the mRNA and protein levels [113]. Combining the effects of CU with those of other substances might result in a ground-breaking treatment for OS. The Chinese Wuhan group has proposed a dual approach for this bone cancer using their hyaluronic acid/silk fibroin photo polymerisable hydrogel with CU-loaded chitosan nanoparticles [114]. According to their research, this substance can both stimulate the growth of MC3T3-E1 murine OB cells in vitro and appear to be able to inhibit the growth of the MG-63 lineage. Consequently, there was an inhibitory impact on tumour cells and, simultaneously, an activation of OB to help regenerate bone [114]. As is already mentioned in this discussion, the PFs have obvious beneficial activities, which have also been demonstrated for bone metabolic interactions. Therefore, using natural polyphenolic substances should also be considered in anti-neoplastic strategies.

**Table 9:** Curcumin’s effects on osteosarcoma and bone neoplasms.

Authors	Journal	Experimental Model	Method of Administration	Type of Effect	Ref.
Aziz et al., 2018	Molecules	In vitro	DK1 was applied on U-2OS and MG-63. Cell cycle analysis, quantitative PCR, and proteome profiling were adapted	DK1 induces apoptosis in human OS through a mitochondrial-dependent signalling pathway; the U-2 OS cell line was more sensitive than other	[108]
Aziz et al., 2021	Pharmaceuticals	In vitro	DK1 was applied on U-2OS and MG-63, microarray gene expression analysis, quantitative PCR, and proteome profiler were elected	DK1 suppressed cell migration, invasion, tube formation, and microvessel formation	[109]
Lu et al., 2020	Molecules	In vitro	L48H37 was applied on U-2 OS and MG-63 in different concentrations. Wound-Healing, Cell Migration and Invasion, Protease, Western Blotting Analysis, and PCR were adapted	L48H37 represses the invasion and migration capabilities of U2OS and MG-63 cells by the suppression of uPA expression and the inhibition of JAK/STAT signalling	[110]
Li et al., 2019	Aging	In vitro	CU was applied on hADSCs. miRNA microarray analysis, Western blot analysis and quantitative RT-PCR analysis were done	CU reduces osteogenesis by stimulating miR-126a-3p and consequently decreasing the WNT/LRP6 pathway	[203]
NEACȘU et al., 2021	Romanian Journal of Materials	In vitro	A nanoparticle of hydroxyapatite-CU was applied on MG63 Molecular and cell analyses were made	This results in a cytotoxic effect on bone cancer cells, activating apoptosis, and increasing the level of AMPK, ARRB1 is associated with a G2/M cell cycle	[111]
Dong et al., 2018	Artificial Cells, Nanomedicine, and Biotechnology	In vitro	ALN-oHA-S-S-CUR micelle on the cell cultures of MDAMB-231, MCF-7	Increased rate of CU release within tumour cells by a reduction-responsive mechanism and CD44 receiving ability	[112]
Wang et al., 2017	Sci. China Mater.	In vitro	CU-NPs were applied on 143B OS cells. Stability test, drug release study, cell analysis, and RTPCR were done	The mRNA and protein expressions of c-Myc and MMP7 are reduced by CU-NPs, which also prevent the metastatic OS 143B cells from proliferating and invading	[113]
Yu et al., 2021	Polymers	In vitro	Chemical hydrogel nanoparticles with CU on MG-63 and ME3T3-E1 cells	Effect of inhibiting the growth of OS cells and promoting the proliferation of pre-OB cells	[114]

### 3.6. Periodontitis and Gum Diseases

Periodontitis is a chronic inflammation sustained by a dysbiosis of the commensal gram-negative bacteria and the host’s immune response. Periodontal disease starts as an acute inflammation of gingival tissue and, if left untreated, leads to the irreversible and progressive destruction of gingival connective tissue and bone. The conventional use of local antimicrobial drugs in the non-surgical treatment of gingivitis and periodontitis can induce the development of bacterial resistance [204,205,206]. Current research focuses on the anti-inflammatory and antioxidant power of some substances so as not to surgically treat the periodontal pockets and inhibit the growth and proliferation of microorganisms. Natural phenolic compounds such as the Q, the CU, and the RSV, due to their anti-inflammatory and antioxidant activities, have been suggested as promising bioactive micronutrients in the treatment of periodontal disease; phenolic compounds appear to play an important role in the modulation of the inflammation reaction and cell differentiation by activating biological pathways [100,207,208]. The Q, the CU, and the RSV could enhance the regenerative properties of periodontal MSCs to lead to bone formation in periodontal bone defects.

#### 3.6.1. Curcumin

Human periodontal ligament stem cells (hPDLSCs) that are derived from the neural crest and endothelial-differentiated hPDLSCs (e-hPDLSCs) produced by an inflammatory stimulus were both examined in the study by Diomede et al. using CU molecules encapsulated in liposomes (CU-loaded liposomes: CU-LIP) (the Lipopolysaccharide was obtained from Porphyromonas gingivalis, LPS-G) (Table 10) [115,209].

Based on their findings, CU-LIP may be able to reduce inflammation and alter the epigenetic patterns in cultured cells. The inflammatory cascade, which includes caspase-1, toll-like receptor-4 (TLR4), MyD88, nuclear factor kappa light chain enhancer of activated B cells (NFkB), NLR Family Pyrin Domain Containing 3 (NLRP3), ROS production, and NFkB are all downregulated in CU-LIP. The study also discovered that LPS-G exposure significantly altered the expression of epigenetic modifiers like P300 and DNA Methyltransferase 1 (DNMT1) [115]. Periodontal tissue regeneration is a hotly disputed topic in the literature, and hPDLSCs seem to be critical players in it [210]. Studies indicate that CU induces the differentiation of MSCs into osteoblasts [211]. Furthermore, CU appears to induce the cell death of osteoclasts [57,212,213]. A study by Xiong et al. in 2020 [116] investigated how CU affected the hPDLSC’s osteogenic differentiation.

This study demonstrated that 0.1 mM of CU is the optimal dose to ensure cell viability and osteogenic differentiation (Table 10) [116]. The CU acts on the phosphatidylinositol-3-kinase (PI3K)/protein kinase B (AKT) signalling pathway and induces an increase in ALP, COL1, RUNX2 and the formation of calcium nodules [116]. The CU, the Q, and the RSV have shown promise in treating periodontitis.

Due to its anti-inflammatory and antibacterial properties, the CU offers a straightforward and non-invasive treatment for periodontal disease. Research on CU, RSV, and Q for gum disease treatments is still limited. Therefore, clinical insights are needed [115].

**Table 10:** Effects of curcumin on periodontitis.

Authors	Journal	Experimental Model	Method of Administration	Type of Effect	Ref.
Diomede et al., 2021	Int J Mol Sci	In vitro	Neural crest-derived human periodontal ligament stem cells phosphate buffered saline MSCBM-CD	CU-LIP helps control the production of ROS and the inflammatory cascade and influences epigenetic mechanisms in vitro	[115]
Xiong et al., 2020	Iran J Basic Med Sci	In vitro	Control group CU 0.001 µM + hPDLSC CU 0.01 µM + hPDLSC CU 0.1 µM + hPDLSC CU 1 µM + hPDLSC CU 10 µM + hPDLSC	Low concentration CU activates the PI3K/AKT/Nrf2 signalling pathway inducing osteogenic differentiation of hPDLSCs	[116]

#### 3.6.2. Quercetin

In the report by Di Cristo et al., the local use of a biomedical membrane composed of Poly (DL-lactic acid) (PLA) nanofibers that are loaded with different amounts of Q has been proposed for the local management of periodontitis (Table 11) [117]. The direct delivery of Q from PLA membranes to the gum pockets enhances the bioavailability and therapeutic effects. The findings presented here show that the PLA-Q membrane can limit biofilm development and control the ROS activity, which are thought to play a role in the pathogenicity of gum disease [214]. Other relevant results regarding the anti-inflammatory properties of the biomedical membrane regard the downregulation in vitro of various inflammatory mediators such as interleukin (IL-1β, IL-6 and IL-8) and TNF-α [117]. The efficacy of Q-loaded membranes was tested by observing the suppression of Pseudomonas Aeruginosa (PAO1) and *Streptococcus mutans*. The efficiency of Q-loaded nanofibers was demonstrated to be sensitive to the pH of the oral environment [215]. The anti-inflammatory activity of PLA-Q was evaluated in vitro and compared to cells treated with lipopolysaccharide (LPS) obtained from *Porphyromonas gingivalis*. The reported results showed that the anti-inflammatory activity of Q-loaded PLA depended on the PLA-Q concentration [117]. PLA-Q fibres can help in periodontitis by controlling the inflammation and oral microbiome maturation and modulation [14,216,217,218]. Modulating microbiota plays a crucial role in periodontitis pathophysiology because, as mentioned above, the disease is caused by destructive host immune responses to pathogenic bacterial species due to oral microbiota dysbiosis [219,220,221,222,223].

### 3.7. Titanium Surfaces Modified with Flavonoids

The great challenge for the success of dental implants is tissue integration, and when a failure occurs it is due to bacterial colonization that promotes infection and inflammatory responses [224,225,226]. The two conditions that may affect peri-implant tissues are mucositis and peri-implantitis; peri-implantitis is the loss of the supporting bone around an implant, whereas peri-implant mucositis is used to explain inflammatory reactions in the mucosa surrounding an implant [224]. In 2008, Ziztmann’s review outlined an epidemiological picture of high incidence: peri-implantitis is found between 28 and 56% of the implants, while peri-implant mucositis—corresponding to gingivitis—reaches 80% [227]; a recent SR of Derks downsizes these data, bringing them to 22 and 43%, respectively [228]. The focus of this research is to create a bioactive surface that could have antibacterial and anti-inflammatory effects and promote osteointegration.

The use of flavonoids may represent an exciting option to modify titanium (Ti) surfaces like that of dental implants. These polyphenolic substances have demonstrated antimicrobial and anti-inflammatory effects [229]. Ti surfaces that are functionalised with flavonoids have an osteopromotive effect, i.e., they encourage the differentiation of hUC-MSCs into osteoblasts by elevating the expression of osteogenic markers, raising alkaline phosphatase activity, and boosting calcium deposition [88,89].

Concerning the use of growth factors, flavonoids are less expansive and simple to applicate in clinical.

Quercetin-The titanium surfaces that are treated with quercetin appear to favour osseointegration by reducing peri-implantitis and implant loss episodes. The Cordoba study [89] evaluated the activity of hUC-MSC and gingival fibroblasts (HGF) in contact with titanium surfaces that were treated with Q. In contrast, in another study in 2018 [118], the same author evaluated the effect of titanium implants that were treated with Q on osteoclasts.

Both studies evaluated the expiration of the material by assessing the liberation of lactate dehydrogenase (LDH: an enzyme produced when the cell membrane is injured). Both studies concluded that titanium surfaces are not toxic to cells [89,118]. These studies showed that there was an overexpression of COL1A1 on titanium surfaces that were modified with flavonoids, while endothelin-1 (EDN1) and interleukin-6 (IL6) were downregulated; this promotes the proliferation of hUC-MSCs. The second study highlighted that Q implant surfaces minimized the activity of osteoclasts [89,118] (Table 12). Therefore, these surfaces encourage cell proliferation and promote osseointegration in the clinical setting and decrease implant failures.

## 4. Conclusions

In recent years, it has become increasingly clear how important knowledge of the products that nature has to offer is for addressing the challenges that medicine faces with new strategies and weapons. There is a growing interest in plant-derived substances that can positively influence human health via a variety of mechanisms that interact on a biochemical level with our organism’s physiological and pathological processes.

This interest has centred on phytochemicals such as phenolic compounds; among these molecules, resveratrol, curcumin, and quercetin have received the most attention.

According to the publications, these substances have a variety of beneficial effects.

Resveratrol, polydatin (a resveratrol glycoside), curcumin, and quercetin all exert an important modulatory effect on bone turnover via various mechanisms of action, such as the stimulation and inhibition of bone cell populations, the inhibition of matrix metalloproteases (MMP-9), the regulation of mesenchymal cell differentiation, and the regulation of gene expression (RANK), all of which intervene in tissue metabolism modulation.

These substances, through these mechanisms, protect tissue from the loss of density associated with pathological processes such as osteoporosis and osteopenia.

Resveratrol and curcumin seem to be effective in the treatment of bone tumours by inhibiting osteosarcoma cell proliferation, while also having anti-metastatic and anti-angiogenic properties. They play an important role in supporting traditional therapies, making them more effective and allowing them to be administered at lower dosages, therefore reducing the risk of side effects, toxicity, and chemoresistance phenomena.

Because these substances inhibit bone catabolism, they are also beneficial to therapies that are aimed at preserving tissue density, such as the use of combination therapies in conjunction with bisphosphonates. Human clinical studies examining the impact on bone health, on the other hand, are scarce, and more research is required.

However, in the field of oral health, the direct administration of quercetin through PLA-Q membranes and curcumin intake have shown a potential effect in limiting the development of oral biofilm and controlling the activity of reactive oxygen species (ROS), which are thought to play a role in the pathogenicity of periodontal disease, as well as inducing an in vitro down-regulation of various inflammatory mediators that are characteristic of periodontal disease. These substances may thus be useful in the treatment of periodontitis by controlling inflammation as well as the maturation and modulation of the oral microbiome, both of which play important roles in the pathophysiology of this disease.

The potential of these substances is not limited to their direct administration or use in combination therapies with traditional drugs. The development of biomaterials that are functionalised with natural molecules represents a significant opportunity for biomedical research. Such as hydroxyapatite-curcumin systems in the treatment of osteosarcomas. The integration of various materials allows their properties to be combined, potentially having synergistic effects on human health. Natural phenolic compounds have health-related functional properties such as antioxidant, antibacterial, and anti-tumour activity, but therapeutic applications are limited due to their low bioavailability and stability in a physiological environment. The polyphenol-functionalisation of implantable materials would allow for more clinical applications.

Biomedical research is looking into material coupling to create a combination of biomaterials that are functionalised with natural molecules to evaluate the potential benefits on biological tissues. Recent research, for example, has demonstrated that coating titanium surfaces with quercitrin has improved the integration of dental implants due to its antioxidant, anti-inflammatory, and antimicrobial properties. This discovery could spur implantology research to promote osseointegration.

The study of natural substances, particularly phenolic compounds, represents a new direction for medical research to better understand their promising potential and make their use applicable in everyday medicine. Further in vivo and in vitro studies on humans are required to shed light on the mechanisms of action and potential applications. In particular, studies to understand the role of resveratrol in the pathophysiology of periodontal disease and quercetin in the pathophysiology of bone tumours are lacking in the literature of the phenolic compounds under study in our research.

More clinical studies on curcumin, resveratrol, and quercetin in the treatment of gum disease are needed, as are human clinical studies examining the influence of curcumin on bone health.

In addition, further studies are needed to define more rigorous and precise administration protocols and dosages, as the studies analysed in this review refer to the administration of varying dosages of these substances. It is also necessary to take into account the adverse effects that these substances may have in the event of overdose or prolonged administration, e.g., curcumin, in high doses, induces cytotoxicity in human osteoblasts, leading to a significant reduction in bone tissue cell density.

Along with the goal of understanding these mechanisms, studies on biomaterials that are functionalised with these substances, that show promise for use in multiple areas of medicine should be conducted concurrently.

## Figures and Tables

**Table 1:** Database search indicators.

**Articles screening strategy**	KEYWORDS: A: “Resveratrol”; B: “Curcumin”; C: “Quercetin”; D: “bone”;
	Boolean Indicators: “A” AND “D”; “B” AND “D”; “C” AND “D”
	Timespan: from January 2017 up to 3 July 2022.
	Electronic Databases: PubMed, Web of Science, Google Scholar, Scopus, Cochrane Library, Embedded

**Table 2:** RSV and polydatin’s effect on osteogenesis and osteoarthrosis.

Authors	Journal	Experimental Model	Method of Administration	Type of Effect	Ref.
Vidoni et al., 2019	Cell Commun Signal	In vitro	HGMSCs cultured with RSV and osteogenic differentiation factors	Up-regulation of autophagy and differentiation of HGMSCs in OB, through the activation of the AMPK-BECLIN1 pathway	[93]
Chen et al., 2019	Biomed Pharmacother	In vitro	HBMSCs and polydatin administration	Osteogenic differentiation and proliferation of HBMSCs were both boosted by PD	[95]
Di Benedetto et al., 2018	Int J Med Sci	In vitro	RSV and Polydatin extracted from Polygonum cuspidatum	Positive effects on bone-related cells and stopping the growth of osteosarcoma cells	[96]
Shah et al., 2019	Pharmacognosy Magazine	In vitro	Alizarin staining for the mineralization assays, total protein content, ALP’s activity, and cell viability assay were used to assess the OB cell proliferation and differentiation potentials of RSV	- Safe over a wider range of concentrations; the EC50 was 72.05 M. - No significantly affect ALP activity at 500 nM or 1 M doses (therapeutic concentration) on hFOB cells. - Cause a dose-dependent rise in the total cellular proteins in hFOB (P 0.05). - Stimulating effect on bone mineralization.	[91]
Abbas et al., 2021	Braz. J. Biol.	In silico and in vitro	In silico: 3D resveratrol structure docked (PyRx free software) with RANKL In vitro: osteoclasts cell from femora and tibia of mice together with M-CSF	RSV binds tightly to RANKL reducing the actin ring formation, decreases the Reactive Oxygen Species (ROS) level, protecting the bone loss	[92]
Li et al., 2018	J Cell Biochem	In vitro study	21-day exposure of hBM-MSCs to hydrogen peroxide (H2O2) (25 μM–100 μM), RSV (5 μM) and nicotinamide (NAM) (10 mM)	SIRT1 is involved in the osteogenesis of hBM-MSCs and antioxidant mechanisms. RSV activates SIRT1, NAM inhibits SIRT1	[94]

**Table 3:** Osteogenesis and antioxidative effect of CU in bone disease.

Authors	Journal	Experimental Model	Method of Administration	Type of Effect	Ref.
Chandra et al., 2000	Free Radic Biol Med	In vitro	hBM-MSCs cultured in an osteogenic medium and treated with NS-J exposed to H_2_O_2_	Curcumin and Boswellia have a strong antioxidative effect and enhance the differentiation of MSC in Ob	[148]
Hatefi et al., 2018	World Neurosurg	Clinical trial	100 patients with Spinal cord injury from a rehabilitation clinic in Ilam City, Iran, between 2013 and 2015	Indicators of BMD significantly improved. Significant differences between the interventional and control groups for the mean BMD of the femoral neck and hip	[57]
Yang et al., 2021	Basic Clin. Pharmacol. Toxicol.	In-vitro study	The hBM-MSCs were treated with 0.01–100 μmol/L curcumin for 7 days	No toxic effects on in vitro maintenance with curcumin amounts of 10 μmol/L or less. CU concentrations of 0.01–1 μmol/L, the hBM-MSCs proliferation significantly increased. CU concentrations of 5 and 10 μmol/L induced cell apoptosis and diminished cell division in hBM-MSCs. Administration of 5 µmol/L of CU lowered the expression of matrix metalloproteinases (MMPs) in hBM-MSCs, enzymes involved in metastatic processes	[98]
Son et al., 2017	Bioorganic & Medicinal Chemistry Letters	In-vitro study	MSCs were exposed to different concentrations of curculactones A or B for 48 h	Induce OB differentiation in MSCs through the osteogenic expression of genes such ALP, OC, Distal-less Homeobox 5, and Runt-related Transcription Factor 2	[99]

**Table 4:** Q effect on HBM-MSCs’ differentiation.

Authors	Journal	Experimental Model	Method of Administration	Type of Effect	Ref.
Torre et al., 2017	Int J Mol Med	In vitro study	Incubation of hBM-MSCs with two types of pomace extracts that are rich in PFs studied by RT-qPCR	Expression of genes involved in OBs differentiation of hBM-MSCs increases	[100]
Bian et al., 2021	BMC Complement Med Ther	In vitro study	qRT-PCR measured the expression of H19, miR-625-5p, BMP-2, osteocalcin and RUNX2; western blotting measured β-catenin protein levels	Q promotes osteogenic differentiation of hBM-MSCs through activation of the H19/miR-625-5p pathway and Wnt/β-catenin	[101]

**Table 5:** Characteristics of the studies about RSV and OP.

Authors	Journal	Experimental Model	Method of Administration	Type of Effect	Ref.
Borsani et al., 2018	BioMed Research International	Study in vitro	HOBs are studied in a medium constituted by osteoblast, antibiotic, and antifungal, at 37 °C, 5% CO_2_. Human OBs were treated with different concentrations of RSV	10 μM RSV promotes human OB growth and differentiation RSV interacts with CGF positively preserving human OB treated with BP	[76]
Li et al., 2019	J Cell Biochem	Study in vitro	hBM-MSCs separated from three patients with OP, cultured in Dulbecco modified Eagle’s cell culture medium, treated with RSV at different concentrations and analysed after 48 h	RSV is essential for proliferation, apoptosis, osteogenesis of hBM-MSCs, and is efficient for treating patients with OP	[102]

**Table 6:** Characteristics of the studies about CU and OP.

Authors	Journal	Experimental Model	Method of Administration	Type of Effect	Ref.
Khanizadeh et al., 2018	Archives of Endocrinology and Metabolism	Double-blind RCT	Sixty PM subjects, control group, alendronate group, alendronate plus CU group; BMDs measured by DXA before and after 12 months of treatment	In alendronate with CU group, decrease of BALP and CTx and rising osteocalcin; increase of BMD indexes in the four areas. No adverse events	[119]
Ali et al., 2017	Cancer Cell Int	Double-blind controlled trial, supplement study	Fifty-seven healthy subjects with low bone density followed either an SM to control low bone density or SM + CU; BMD of heel, small finger and upper jaw assessed at the start and weeks 4, 12, 24	In CU-group, increase of BMD of the heel at week 12 and 24, of small finger and upper jaw at week 24. No safety and tolerability issues	[120]

**Table 7:** Characteristics of the study about Q and bone mineralization biomarkers.

Authors	Journal	Experimental Model	Method of Administration	Type of Effect	Ref.
Hassan et al., 2018	Advances in Pharmacology and Pharmacy	Double-blind RCT	Forty patients with type 2-DM, 45–50 years, Q group vs. placebo; Serum calcium, osteocalcin, [25(OH)D] level measured at inclusion and 3 months	Increase in serum [25(OH)D] and calcium in the Q group	[121]

**Table 8:** Multiple effects of RSV on bone neoplasms.

Authors	Journal	Experimental Model	Method of Administration	Type of Effect	Ref.
Gao et al., 2017	Oncol Let	In vitro	Ten oligostilbenes and human cancer cell lines	Human bone cancer cells show antiproliferative action when exposed to RSV	[103]
Xie et al., 2017	Oncotarget	In vitro	RSV and OS cells	RSV promotes apoptosis and inhibits U2-OS cell motility, invasion, glycolysis, and proliferation	[104]
De Luca et al., 2022	Pharmaceuticals	In vitro	RSV was applied to OS cell lines at doses of 0–10–25–100 µM	decrease in tumour cell proliferation after treatment with RSV-doxorubicin (DOX) or RSV-cisplatin (CIS)	[105]
Lama et al., 2022	Pharmaceuticals	In vitro	PD on Polycaprolactone Nanofibers	PD promoted the proliferation of MSCs while inducing cell toxicity in Saos-2 cells	[106]
Luce et al., 2021	Oxid Med Cell Longev	In vitro	PD on Saos-2	PD pretreatment may boost radiotherapy’s efficacy	[107]

**Table 11:** Effects of quercetin in periodontitis.

Authors	Journal	Experimental Model	Method of Administration	Type of Effect	Ref.
Di Cristo et al., 2022	Molecules	In vitro	PLA (0.7 mol% L-isomer and polydispersity), Q Chloroform (CHL), N,N-Dimethylformamide (DMF), ethanol and acetone, *Streptococcus mutans* (ATCC^®^ 25,175), *Pseudomonas aeruginosa* PAO1 (ATCC^®^ BAA-47TM), LPS-G, immortalised human gingival fibroblast	PLA-Q membrane can limit biofilm maturation and exerts antioxidant and anti-inflammatory effects	[117]

**Table 12:** Studies about titanium surfaces modified with Q.

Authors	Journal	Experimental Model	Method of Administration	Type of Effect	Ref.
Cordoba et al., 2015	Adv. Healthcare Mater.	In vitro	The surface of titanium discs was aminosilanized with 3-aminopropyl) triethoxysilane (APTES) and then the Q was covalently bound, and the activity was evaluated with hUC-MSC and HGF cells	1. Flavonoid-modified titanium surfaces are not toxic to cells as there was no production of LDH. 2. On the flavonoid-modified titanium surfaces, there was an overexpression of COL1A1, while EDN1 and IL6 were downregulated. 3. The metabolic activity of hUC-MSCs grown on APTES surfaces had high metabolic activity.	[89]
Cordoba et al., 2018	International Journal of Molecular Sciences	In vitro	RAW264.7 cells	Q implant surfaces reasonably reduced the expression of osteoclast-related genes in vitro (Trap, CalcR, Ctsk, H + ATPase, Mmp9)	[118]

## Data Availability

Not applicable.

## Abbreviations

AKT	protein kinase B
ALN-oHA-S-S-CU	poly-alendronate-ialuronane-S-S-curcumin
BMD	bone mineral density
CGF	Concentrated Growth Factors
CTX	C-Terminal Telopeptide
CU	Curcumin
CU-NPr	nanoparticles coated with curcumin
CU-Lip	Curcumin-loaded liposomes
DK1	(Z)-3-hydroxy-1-(2-hydroxyphenyl)-3-phenylprop-2-en-1-one
DNMT1	DNA Methyltransferase 1
e-hPDLSC	endothelial-differentiated hPDLSC
ECM	extracellular matrix
FDA	Food and Drug Administration
GRAS	General Recognition and Safety
GUSB	β-glucuronidase
hADSCs	human adipose-derived mesenchymal stem cells
hBM-MSCs	mesenchymal stem cells derived from human bone marrow
hPDLSC	Human Periodontal Ligament Stem Cell
hUC-MSC	Human Umbilical Cord- derived Stem cell
L48H37	1-ethyl-3,5-bis((E)-3,4,5-trimethoxybenzylidene)piperidin-4-one
LPS-G	Lipopolysaccaride obtained from Porphyromonas gingivalis
MSC	Mesenchymal Stem Cell
NFkB	Nuclear factor kappa light chain enhancer of activated B cells
NLRP3	NLR Family Pyrin Domain Containing 3
NS-J	combination of CU, ginger, vitamin D, L. rhamnosus, and Boswellia extract
OB	osteoblasts
OP	osteoporosis
OPG	osteoprotegerin
OS	Osteosarcoma
PAO1	Pseudomonas Aeruginosa
PFs	phenolic compounds
PI3K	Phosphatidylinossitol-3-kinase
PLA	Poly DL-lactic acid
PM	postemenopausal
PVL	Panton-Valentine Leukocidin toxin
Q	Quercetin
RANKL	RANK Ligand
RCT	randomised control trial
ROS	Reactive Oxygen Species
RSV	Resveratrol
SIRT1	Activates Sirtuin 1
TLR4	Tool-like receptor 4
TNF	Tumour Necrosis Factor
[25(OH)D]	25 vitamin D hydroxylase
